# Wi-CHAR: A WiFi Sensing Approach with Focus on Both Scenes and Restricted Data

**DOI:** 10.3390/s24072364

**Published:** 2024-04-08

**Authors:** Zhanjun Hao, Kaikai Han, Zinan Zhang, Xiaochao Dang

**Affiliations:** 1 College of Computer Science and Engineering, Northwest Normal University, Lanzhou 730070, China; haozhj@nwnu.edu.cn (Z.H.); 2021212121@nwnu.edu.cn (Z.Z.); dangxc@nwnu.edu.cn (X.D.); 2 Gansu Province Internet of Things Engineering Research Center, Lanzhou 730070, China

**Keywords:** WiFi sensing, cross-domain, few-shot learning, human activity recognition

## Abstract

Significant strides have been made in the field of WiFi-based human activity recognition, yet recent wireless sensing methodologies still grapple with the reliance on copious amounts of data. When assessed in unfamiliar domains, the majority of models experience a decline in accuracy. To address this challenge, this study introduces Wi-CHAR, a novel few-shot learning-based cross-domain activity recognition system. Wi-CHAR is meticulously designed to tackle both the intricacies of specific sensing environments and pertinent data-related issues. Initially, Wi-CHAR employs a dynamic selection methodology for sensing devices, tailored to mitigate the diminished sensing capabilities observed in specific regions within a multi-WiFi sensor device ecosystem, thereby augmenting the fidelity of sensing data. Subsequent refinement involves the utilization of the MF-DBSCAN clustering algorithm iteratively, enabling the rectification of anomalies and enhancing the quality of subsequent behavior recognition processes. Furthermore, the Re-PN module is consistently engaged, dynamically adjusting feature prototype weights to facilitate cross-domain activity sensing in scenarios with limited sample data, effectively distinguishing between accurate and noisy data samples, thus streamlining the identification of new users and environments. The experimental results show that the average accuracy is more than 93% (five-shot) in various scenarios. Even in cases where the target domain has fewer data samples, better cross-domain results can be achieved. Notably, evaluation on publicly available datasets, WiAR and Widar 3.0, corroborates Wi-CHAR’s robust performance, boasting accuracy rates of 89.7% and 92.5%, respectively. In summary, Wi-CHAR delivers recognition outcomes on par with state-of-the-art methodologies, meticulously tailored to accommodate specific sensing environments and data constraints.

## 1. Introduction

Human activity recognition (HAR) plays a pivotal role in emerging Internet of Things (IoT) technologies, encompassing domains such as smart healthcare, smart homes, and user identification [1,2]. Numerous HAR systems exist, including camera-based approaches [3], wearable sensor-based methods [4], radio frequency-based techniques [5,6], ultrasonic-based solutions [7], and FMCW-based methodologies [8,9]. Despite the commendable recognition performance demonstrated by these HAR systems, practical deployment poses several challenges, such as privacy and security concerns, high equipment costs, limited sensing distances, and installation or wearing requirements.

HAR based on WiFi Channel State Information (CSI) has emerged as a focal point in intelligent sensing research. In comparison to other sensing technologies, WiFi sensor devices offer advantages in terms of cost-effectiveness, ubiquity, security, and ease of deployment. CSI, being highly responsive to human motion, provides detailed amplitude and phase information across subcarriers in the frequency domain. Leveraging these technical merits of WiFi, researchers have proposed device-free human sensing applications utilizing WiFi CSI, including indoor localization [10], intrusion detection [11], vital sign monitoring [12], and gesture recognition [13].

Meanwhile, numerous deep learning-based studies [14] have made significant strides in this domain, particularly in understanding the pattern relationships between CSI patterns and activity types. However, WiFi signals are susceptible to absorption, diffraction, reflection, or scattering phenomena during propagation, resulting in a strong coupling relationship between CSI and environmental factors beyond human actions. The CSI patterns elicited by the same action in varying environments or under different conditions may exhibit disparities. While high accuracy can be achieved if HAR models are trained and tested in identical locations, their performance drastically declines when confronted with new activity classes, users, or scenarios, thus presenting a cross-domain challenge [15]. To tackle this issue, numerous studies have proposed WiFi-based cross-domain HAR approaches. However, some methodologies exhibit inherent limitations. Moreover, in larger environments featuring multiple WiFi devices, dynamic sensing device selection could significantly enhance sensing accuracy and efficacy, further bolstering HAR applications.

To address the aforementioned issues, we have designed Wi-CHAR, a WiFi-based Cross-domain Human Activity Recognition system utilizing few-shot learning. Wi-CHAR comprises two key modules. Firstly, focusing on scene analysis, it utilizes access points (APs) and WiFi-enabled sensors to establish transmission pairs. We propose a method for dynamically selecting the optimal sensing receiver device based on the individual’s location. The fundamental idea is to utilize multiple WiFi device transceiver pairs to comprehensively select sensing devices tailored to the specific environmental layout. Secondly, Wi-CHAR prioritizes constrained data, thereby avoiding cross-domain pattern alterations by employing similarity metrics instead of CSI patterns, to some extent resolving the cross-domain challenge. By capturing a small volume of action data for few-shot learning, Wi-CHAR can detect human activities across multiple environments without necessitating retraining in a new domain, thus mitigating data labeling and training burdens, achieving generalization. A perceptual recognition model can be derived from a limited number of training samples using a few-shot learning algorithm, thereby enhancing system robustness through the amalgamation of diverse samples and aiding the recognition model in delineating clearer boundaries. Additionally, this paper proposes a method to enhance the structure and fortify the noise immunity of the prototype network. Conventional prototype networks often exhibit poor noise immunity, leading to decreased model accuracy in the presence of noise interference. However, by reassigning feature embeddings to mitigate noise impact, Wi-CHAR effectively improves noise immunity, thereby enhancing overall model performance.

The system’s performance is evaluated by conducting a series of experiments on its own human activity datasets under different conditions. Based on this, the system’s performance is analyzed to recognize new user behaviors and scenarios under fewer sample conditions. Comparative experiments are also conducted in this paper to verify the reliability and robustness of the system for activity recognition with limited training samples. This paper also performs performance evaluations on the public datasets WiAR [16] and Widar 3.0 [15]. The experimental results show that the system can recognize common human activities with high accuracy based on the available support sets. In summary, this paper makes the following contributions:

(1) Consider the problem of sensing restricted data. Wi-CHAR can be used in new domains using only a small number of labelled samples, eliminating the need to retrain new models.

(2) Take into account the sensing scenes, design an adaptation model for partial area sensing capability decrease. This can obtain higher quality data over a greater sensing region and improve the human activity recognition effect.

(3) We propose a prototype network structure Re-PN to improve the noise immunity performance of the system. Compared with the basic prototype network, the average performance of the proposed method is improved by 12%.

The rest of the paper is organized as follows. Section 2 describes the related work. Section 3 details the process of implementing the system. Section 4 provides the analysis and evaluation of the experimental results, and Section 5 concludes the paper.

## 2. Related Work

### 2.1. Non-Few-Shot Learning with WiFi HAR

Fine-grained CSI has been widely used for human motion detection in the past few years. CrossSense [17] utilizes simulated CSI samples from the target environment to retrain the recognition model, thereby enhancing performance in new environments. Widar 3.0 [15] introduces a generalized deep learning model for cross-domain gesture recognition, requiring only one-time training and adaptable to diverse data domains. Wang et al. [13] introduced SS-GAN and ST-GAN, which augment the training sample set by generating virtual samples to address gesture recognition challenges in novel scenarios. WiDIGR [18] uses a two-dimensional Fresnel zone to eliminate the effect of walking directly on the signal spectrogram. CeHAR [19] was proposed as a parameter-free dual-feature fusion method with compact fusion of CSI amplitude and phase features. Sheng et al. [20] used a trained source domain model as a pre-trained model in a new scene. Zhang et al. [21] proposed a Dense-LSTM that expanded the training datasets by eight CSI transform methods and achieved about 90% accuracy in adapting to recognize new individual activities. WiLCA [22] implemented a cross-domain authentication system using a small amount of data. Sun et al. [23] conducted research on WiFi-based human motion detection through walls, using an iterative adaptive approach to improve Doppler resolution and further extend the potential of WiFi for through-wall sensing applications. Zhou et al. [11] combined the Back Propagation Neural Network (BPNN), the Adaptive Genetic Algorithm (AGA), and CSI tensor decomposition to improve data processing while obtaining high indoor positioning accuracy.

All these approaches aim at detecting human motion within the sensing range of Wi-Fi devices. WiFi-based sensing systems have very large sensing ranges and fuzzy sensing boundaries. These methods are not friendly to additional training for each new domain. The Wi-CHAR platform in this paper is based on an accurate sensing boundary model for device selection. It achieves higher accuracy in cross-domain sensing that is robust to different environments.

### 2.2. Few-Shot Learning with WiFi HAR

Many recent works use few-shot learning, such as WiLISensing [24], a location-independent, limited-data human activity recognition system. Inspired by relational networks, ML-DFGR [25] proposed a WiFi gesture recognition system that is robust to new users and environments due to its transferable similarity evaluation capability. AFSL-HAR [26] achieved significant performance in identifying new categories by fine-tuning the model parameters with a small number of samples. AirFi [27] proposes that the domain generalization effect of perception can be further improved by using the method of few-shot learning. MatNet-eCSI [28] proposes a neural network with enhanced external memory to improve environmental robustness through one-shot learning. MetaSense [29] adopts a few-shot learning framework, enabling deep mobile sensing methods to rapidly adapt to new users and new devices. RF-Net [6] employs a metric-based meta-learning framework to achieve cross-environment HAR using two pairs of WiFi devices; however, RF-Net’s cross-domain performance is limited. OneFi [30] adopts a single-sample learning framework to recognize unseen gestures, yet this requires four receivers to convert existing gestures into virtual gestures, a process that demands intricate knowledge.

Collecting a large amount of data can be very expensive and, in some cases, even impossible. Therefore, this paper is inspired by few-sample learning to build models using fewer samples to reduce the cost of model building and improve scalability in new environments. Wi-CHAR can be used in new scenarios using only a small number of labeled samples without the need to train a new model.

## 3. System Design

In this section, we present the system design. Firstly, we describe the overall architecture of the framework. Subsequently, we provide a comprehensive overview of the dynamic selection method, data processing, feature extraction, and the enhanced prototype classification network for receiving devices across multiple links within this system. Finally, we briefly outline the approach for implementing the training of the activity recognition model.

### 3.1. Overall System Architecture

**Figure 1:**
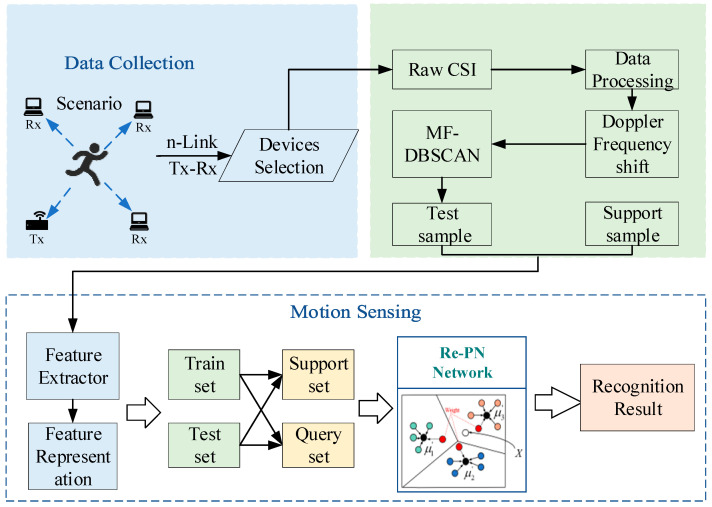
Wi-CHAR system framework.

Figure 1 presents an overview of the Wi-CHAR framework, divided into two main components: the data processing part and the motion sensing part. In the data acquisition and processing stage, it verifies the suitability of device arrangement in the scene, ensures the proper functioning of Tx–Rx pairs, and selects devices based on specific locations. The data from the most suitable receiving device is utilized for recognition. During the activity recognition phase, input features of the PN model are constructed to train the sensing model. Recognition results can be further obtained by adjusting the weights assigned to the PN.

### 3.2. Dynamic Selection of Rx in n-Links

The prevalence of WiFi sensor devices in indoor environments makes the optimal solution choice possible. Not all Tx–Rx pairs are equally good at sensing because the position and orientation of the target relative to the Tx–Rx pair affect the sensing accuracy, and the sensing recognition under a single transmit–receive link suffers from a position-dependent problem. Sensing-Signal-to-Noise-Ratio (SSNR) [31] can quantify the sensing capability. Assuming that the settings of the WiFi transceiver pair are known and the distance from the sensed target to the transmitter and receiver is the Line of Sight (LoS) path length, then we have:

(1) $$SSNR\propto \frac{{r_{D}}^{2}}{{\left(r_{T}r_{R}\right)}^{2}},$$

where *r_D_* is the distance between the transmitter and receiver, i.e., the path length of the LoS, *r_T_* and *r_R_* are the distances from the target to the transmitter and receiver, respectively. In a real indoor environment, there are many other objects on reflection. To extend the sensing coverage model to a multipath-rich environment, Equation (2) is used to represent the power variation due to multi-path:

(2) $${\left(r_{T}r_{R}\right)}_{b}=\sqrt{\frac{K}{4\pi (\gamma (P_{LoS}+\Delta P)+b)SSNR_{min}}},$$

where *γ* is the slope of the linear curve, *b* is a constant, *γ*, *K*, and *b* have a fixed value for each pair of transceiver, and $P_{LoS}$ is the static path signal power. It is shown that the SSNR is related to the distance from the target to the transceiver device and the distance of the transceiver setup. The dynamic receiver device selection step is as in Algorithm 1. Removal of receivers with poor sensing capability according to the above SSNR and iteration to obtain the optimal receiver location.

**Figure 2:**
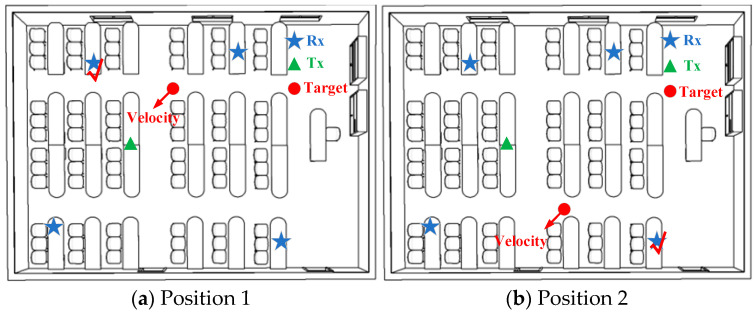
WiFi receiving device selection for targets in different positions.

To verify the device selection model, the dynamic selection of sensing devices is performed after determining the area. The best sensing–receiving device within a certain area is obtained, as shown in Figure 2. The data are obtained to pave the way for later activity recognition.

### 3.3. Data Processing and Feature Extraction

CSI has finer subcarrier-level granularity than RSSI [32] and is easily accessible through commercial WiFi devices. WiFi CSI has multi-path propagation and can be represented as a linear superposition of all paths, including noise $\left(H_{n}(f,t)\right)$, dynamic paths $\left(H_{d}(f,t)\right)$, and static paths $\left(H_{n}(f,t)\right)$:

(3) $$H(f,t)=|H_{s}(f,t)|e^{-j\theta_{s}}+|H_{d}(f,t)|e^{-j\cdot 2\pi \frac{d(t)}{\lambda }}+|H_{n}(f,t)|e^{-j\theta_{n}},$$

where $\theta_{s}$ and $\theta_{n}$ denote the amplitudes of the static path signal and noise, respectively. Doppler frequency shift (DFS) can be obtained after a short-time Fourier transform (SFFT) of the channel frequency response of the CSI signal as follows:

(4) $$f_{D}=-\frac{1}{\lambda }\frac{d}{dt}d(t),$$

where λ is the wavelength and d(t) is the length of the reflection path. The CSI after time-frequency analysis can be expressed as the Doppler shift D(f,t):

(5) $$D(f,t)\approx H_{s}(f)+\sum_{k\in H_{d}}\alpha_{k}(t)B(f_{D_{k}}(t))+H_{n}(f),$$

**Figure 3:**
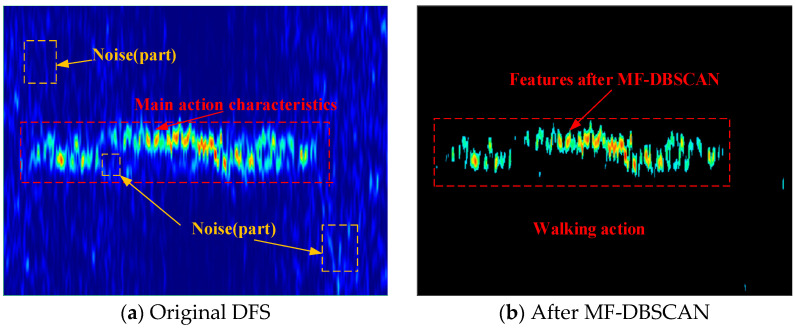
The effect of MF-DBSCAN implementation.

**Algorithm 1** Dynamic Device (Rx) Selection Algorithm**Input:** Tx and Rxs position PTx,PRx{1,…,N}, Rx number N, Parameters $r_{T},r_{R},r_{D}$, Position of the target x at t:$P^{t}$, The static path signal power of Rx at moment t: $P_{Los}^{t}$. **Output:** Res (selection result) of the Rxs selected at time t. //First exclude Rx outside the induction zone. 1: Angle $A^{t}$ of the target at position $P^{t}$ and $r_{T}$ with Tx; 2: **for** i in {1,…,n} **do** 3:   Angle $A_{i}^{t}$ of the target at position $P^{t}$ and $r_{R}$ with Rx; $4:\;\;r_{D}^{2}/{\left(r_{T}r_{R}\right)}^{2}\to SSNR$;  //Preliminary SSNR. 5: **end for** 6: **for**
j **in** {1,…,Res_Rx} **do** 7:   Get position relationship →SSNR{j};//Candidates. 8:   Computation ${\left(r_{T}r_{R}\right)}_{b}$ and $(\gamma (P_{Los}^{t}+\Delta P)+b)SSNR_{min}$; 9:   An equivalent Rx←Res($P^{t}$); 10: **end for** 11: Select an optimal Rxs with direction: Res. where $B(f_{D_{k}}(t))$ is the window function for cutting the new number segment of interest. The raw CSI data often contains noise, and hardware devices may introduce offsets that can adversely affect experimental results when used directly. In this paper, upon acquiring the raw CSI data, we initially denoised the CSI signal using a high-pass filtering method, followed by PCA for extracting principal component feature data. Active samples were then extracted using a threshold-based segmentation method. Finally, a short-time Fourier transform (STFT) was performed to extract the discrete Fourier spectrum (DFS) of the action signal. This paper uses the MF-DBSCAN clustering algorithm to cluster the obtained Doppler spectrograms and correct or remove the anomalies twice. Compared with the K-means algorithm, the DBSCAN algorithm does not need to specify the number of classes for clustering in advance. It can be applied to a wider range of data with arbitrary shapes and can also find outliers. In our experiments, we achieved improved results with reduced arithmetic processing for specific sensing data. The MF-DBSCAN algorithm is detailed in Algorithm 2, and the clustering results are illustrated in Figure 3. As CSI samples for different actions may vary in length, it is crucial to normalize the sample lengths to a fixed duration.
**Algorithm 2** MF-DBSCAN Algorithm**Input:** Raw DFS data. Output: Pre-processed DFS (MF-DBSCAN results) 1: Kernel density estimation, eps_list; mathematical expectation, Minpts_list; raw data label_num; 2: **do** 3:  Splitting by minimum interval eps_list, Minpts_list; 4:  Calculate number of clusters ζ according to eps_list, Minpts_list; 5:   **if** ζ=lable_num Calculation contour coefficient λ; 6:     Compare λ, select maximum λ_Max; 7:     Get MinPts and Eps corresponding λ_Max; 8:   Get the globally optimal MinPts, Eps: 9:  **else** marked as noise; 10: **until** no tagged objects.

In existing few-shot learning studies, two types of feature embedding models are commonly used, including the four-layer convolutional network structure (Conv4) and ResNet18 [33]. The ResNet18 model has a deeper network structure than Conv4 and has significant advantages in generalization performance, so ResNet18 convolutional architecture is used as the action segmentation post-backbone of the feature extractor to extract the feature data of the segmented DFS sequence. Let $f_{\theta }$. be the feature extraction network, where θ is the learnable parameter. Given the input data x, the feature representation $z=f_{\theta }\left(x\right)$.

### 3.4. Re-PN Module

This paper aims to improve the generalization of the classifier obtained by training with a small amount of data. The prototype network (PN) is the focus of the metric learning network, which is simple and effective, avoiding the complexity of recursive networks and reducing memory requirements. All data samples in the training and test sets will be divided into support and query sets. Suppose there is a support set of N labeled samples $S=\{(x_{1},y_{1}),\ldots ,(x_{N},y_{N})\}$, where $x_{i}\in {\mathbb{R}}^{D}$ is the D-dimensional feature vector of the samples and $y_{i}\in \{1,\ldots ,k\}$ is the corresponding label. $S_{k}\in S$ denotes the set of samples labeled as class k. The D-dimensional original data are first mapped to the M-dimensional embedding space θ. For the support set, all $\left|S_{k}\right|$ sample images of the same class are extracted by the neural network feature mapping function $f_{\theta }$ features. For the query set sample $\overset{⌢}{x}$, it is projected into the same feature embedding space $f_{\theta }(\overset{⌢}{x})$ as the support set sample, and the distance is measured by clustering prototypes $\mu_{k}$ with each class of the query set and giving a prediction of the class label $\overset{⌢}{y}$ to which it belongs to:

(6) $$p_{\theta }(\overset{⌢}{y}=k|\overset{⌢}{x})=\frac{\exp(-d(f_{\theta }(\overset{⌢}{x}),\mu_{k}))}{\sum_{k^{\prime }}\exp(-d(f_{\theta }(\overset{⌢}{x}),{\mu_{k}}^{\prime }))},$$

where $\mu_{k}^{\prime }$ denotes the prototype of the action type. The optimization of the prototype network model is achieved by minimizing the negative log probability of correct labels by the gradient descent method:

(7) $$J(\theta )=-\log p_{\theta }(\overset{⌢}{y}=n|\overset{⌢}{x}),$$

where n is the true label of the training sample. The updated loss function of the prototype network model is expressed as:

(8) $$J\leftarrow J+\frac{1}{\lambda n}[d(f_{\theta }(\overset{⌢}{x}),\mu_{k}^{\prime })+\log\sum_{k^{\prime }}\exp(-d(f_{\theta }(\overset{⌢}{x}),\mu_{k^{\prime }}^{\prime }))],$$

**Figure 4:**
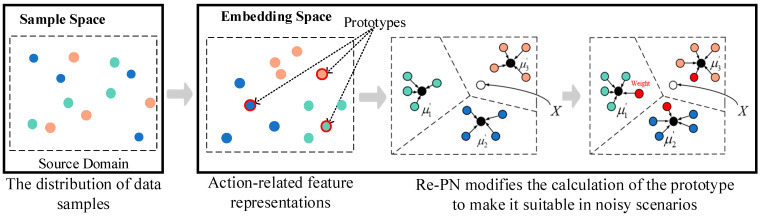
Schematic diagram of activity recognition based on few-shot learning.

CSI data obtained in real-world scenarios often contain significant noise and interference, leading to a notable degradation in the accuracy of traditional PN models under such conditions. Wi-CHAR introduces a method to enhance the PN structure, termed Re-PN, aiming to bolster its noise immunity performance through a reassignment approach. Algorithm 3 outlines the Re-PN methodology, wherein adjustments are made adaptively. This adaptive adjustment endows Re-PN with the capability to differentiate between correct and noisy data samples. It emphasizes the importance weight of correct samples while simultaneously mitigating the interference caused by potential noisy samples on the feature prototype representations. The schematic diagram illustrating Re-PN is depicted in Figure 4, given a test set T of samples $x_{j}^{T}$, a support set $S={\left\{(x_{i}^{S},y_{i}^{S})\right\}}_{i=1}^{M}$, and a query set $Q={\left\{(x_{j}^{S},y_{j}^{S})\right\}}_{i=1}^{N}$. For the support set feature embedding $f_{\theta }(x_{i})$, the improved design introduces a weight parameter $\alpha_{i}$ to measure the degree of influence of a certain sample $x_{i}$ feature embedding of the support set on the feature prototype computation. The feature embedding computation based on the reassignment method network model is expressed as:

(9) $$\mu_{k}^{\prime }=\frac{\sum_{i=1}^{\left|S_{k}\right|}\alpha_{i}f_{\theta }(x_{i})}{\sum_{i=1}^{\left|S_{k}\right|}\alpha_{i}},$$

where $S_{k}$ denotes all similar images belonging to the category k in the support set.

(10) $$\alpha_{i}=\frac{1}{d(f_{\theta }(x_{i}),\frac{1}{|S_{k}|-1}\sum_{j=1,j\neq i}^{\left|S_{k}\right|}f_{\theta }(x_{j}))},$$

where d(⋅) is the distance metric function. The predicted probability distribution of the test sample $x_{j}^{T}$ over each class is calculated by Equation (7). Replacing the test set Q with the query set T in the training phase, the loss can be obtained by the central loss function as follows:

(11) $$L_{c}=\frac{1}{2}\sum_{i=1}^{m}||x_{i}-c_{yi}|{|}_{2}^{2},$$

where $c_{yi}$ denotes the feature embedding center of the $y_{i}$ category sample and $x_{i}$ denotes the feature before the fully connected layer. The final loss function of the model is:

(12) $$Loss=-\log({\hat{y}}_{jk}^{T})+\eta L_{c},$$

where η is the hyperparameter and is taken as η=1 in the experiment. We use an episode-based strategy to train the Re-PN model. Finally, the loss function of Equation (12) is calculated. The training of the model is implemented using the Adam optimization algorithm to update the parameters of the model, and the learning rate parameter $L_{r}$ is updated using the cosine annealing learning rate update strategy:

(13) $$L_{r}=L_{r}\times \frac{1}{2}(1+\cos(\pi \frac{epoch}{\max\_epoch})).$$

where epoch is the number of current iterations and max_epoch is the total number of training sessions. The above process is repeated until the parameters of the network model do not change much.
**Algorithm 3** Re-weighting prototypical network model (Re-PN model)**Input:** Training set $P=\{(x_{1},y_{1}),\ldots ,(x_{N},y_{N})\}$, Number of categories N contained in the support set, K is the number of classes in the training set. **Output:** Re-PN Loss J of Classifier Model. 1: $V\leftarrow Rs(\{1,\ldots ,K\},N_{C})$;      //Few-shot task set. 2: **for** k **in** $\left\{1,\ldots ,N_{C}\right\}$ **do** 3:    $S_{k}\leftarrow Rs(P_{Vk},N_{S})$;       //Select support set. 4:   **for**
i **in** $S_{k}$ **do** 5:    Calculate Equation (10) $\alpha_{i}$;  // Get weight parameters. 6:   **end for** 7:   Calculate $\mu_{k}^{\prime }=\sum_{i=1}^{\left|S_{k}\right|}\alpha_{i}f_{\theta }(x_{i})/\sum_{i=1}^{\left|S_{k}\right|}\alpha_{i}$ feature prototype; 8: **end for** 9: Loss J←0; 10: **for** c
**in** $\left\{1,\ldots ,N_{C}\right\}$ **do** 11:   $Q_{k}\leftarrow Rs(P_{Vk}\backslash S_{k},N_{Q})$;  //Select query set. 12:  **for** (x,y) **in** $Q_{k}$ **do**     //Calculate losses and update model parameters. 13:   Calculate losses $L_{p},L_{c}$; 14:   update Loss$J\leftarrow J+L_{p}+L_{c}$. 15:   **end for** 16: **end for**

## 4. Experiments and Performance Analysis

In this section, we first present the experimental setup. Then, the effectiveness of Wi-CHAR on owned and public data is evaluated in intra-domain and cross-domain scenarios. The performance of different hyperparameter settings is also compared with the most advanced HAR systems to validate system performance.

### 4.1. Experimental Setup

A TP-LINK AX3000 router was used as a transmitter (Tx), and multiple Google Nexus 6P smartphones with Nexmon [34] framework and Thinkpad X201i devices with Intel 5300 Tools [35] were used as receivers (Rx) to collect CSI samples of human activity during the experiments.

In order to systematically evaluate the performance of Wi-CHAR, this study was conducted with several subjects. In the movement monitoring phase, a variety of common postures were evaluated in this paper, i.e., sitting still, walking and standing up, and sitting down. Sudden states such as falls were measured. Data were available for six categories of human activities, as shown in Table 1.

**Figure 5:**
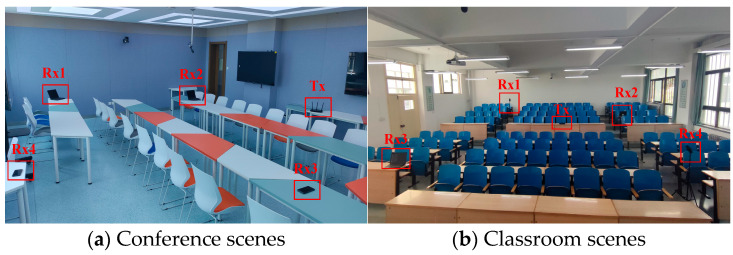
Scenarios for collecting human activity datasets.

The samples were collected in three scenarios: a conference room and a large classroom, as depicted in Figure 5. A total of six subjects (three male and three female) participated in the experiment, and we also examined the impact of their physical parameters (e.g., height, weight, age) on the experiment. Wi-CHAR necessitates at least two receivers in each region to capture the complex changes in path velocity induced by the target’s motion. Initially, three thousand movement data points were generated to form the sample set. Subsequently, only a small number of data samples were collected within the experimental scenario to facilitate motion sensing. Furthermore, the performance of the Re-PN model was validated on the public datasets Widar 3.0 [15] and WiAR [16]. No additional restrictions were imposed on the participants during the experiments. Each environment was equipped with a camera to record all target activities as a reference for the experiment. The training and testing phases were conducted on a Windows desktop featuring an Intel Core i9-10700kF CPU, 24GB RAM, NVIDIA GeForce GTX 3080ti GPU, and PyTorch-1.8.0 framework.

### 4.2. Performance Overview

To accurately and comprehensively evaluate the performance of Wi-CHAR, numerous experiments were conducted under various conditions. Initially, the effectiveness of the Wi-CHAR system within the same domain was tested. Subsequently, the system’s performance with new users, new scenarios, and different datasets was assessed. In each cross-domain experiment, only one domain factor was altered.

This study primarily relies on recognition accuracy as an evaluation metric. It signifies the probability of correctly recognizing an action sample and is calculated using the equation:

(14) $$Accuracy=\frac{TP+TN}{TP+TN+FP+FN}\times 100\%.$$

where TP and FP represent true positive and false positive, respectively. TN and FN represent true negative and false negative, respectively. TP+TN is the number of correctly identified signal samples, and the denominator is the number of all samples tested. The higher Accuracy it is, the better the performance of our system.

#### 4.2.1. Evaluation within the Intra-Domain

**Figure 6:**
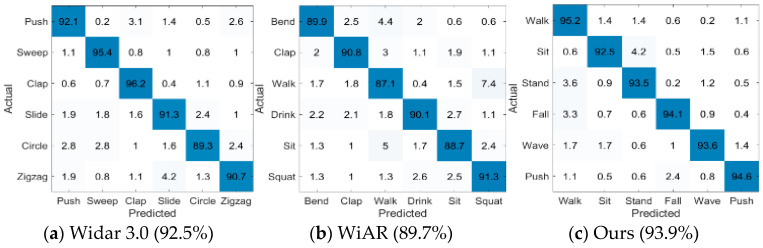
Confusion matrix calculated in three action datasets.

We first evaluate the performance of the proposed method traditionally, i.e., all CSI sample sets are from activities performed by a single user in the same scenario. Figure 6 shows the confusion matrix evaluated in the same domain on Widar 3.0, WiAR, and our own datasets. The proposed system, Wi-CHAR, achieves 93.9%, 92.5%, and 89.7% accuracy on its own datasets, Widar 3.0 and WiAR, respectively. The Euclidean distance metric is used in the experiments, and each action category in the support set contains only five samples. This section uses 80% of the remaining data as the training data and 20% as the test set.

#### 4.2.2. Cross-Scene Recognition Effect

**Figure 7:**
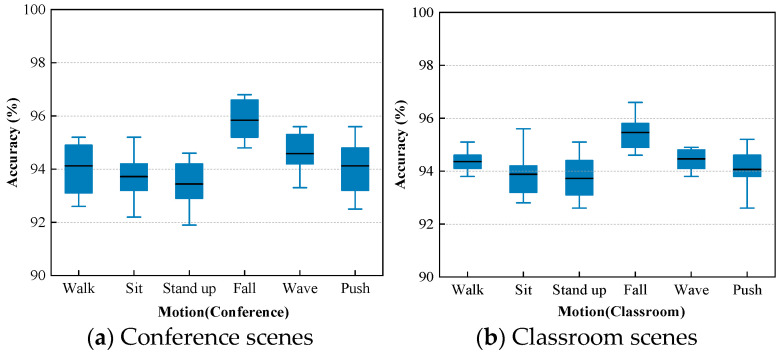
Recognition accuracy of actions in different environments.

Empty rooms were chosen as the source domain, while conference rooms and large classrooms were designated as the target domains. Each experiment was repeated 10 times, and the objective evaluation results are depicted in Figure 7. The average accuracy of practical actions on our own data surpasses 93%, with the highest accuracy exceeding 96% (five-shot).

In the Widar 3.0 datasets, M1, M2, and M3 represent the lounge, conference room, and laboratory, respectively, while W1, W2, and W3 denote the classroom, office, and hall, respectively. The experimental results obtained are presented in Table 2 and Table 3, respectively. As observed in Table 2, the additional scene data collected also exhibits superior recognition rates with Wi-CHAR, further highlighting the system’s cross-scene capability.

#### 4.2.3. Cross-User Recognition Effect

To evaluate the cross-user performance of Wi-CHAR, this study trained the model using CSI samples collected from one user and tested the system’s performance using CSI activity samples from other users (u1, u2, u3, u4, u5). One of the sixteen experimenters (p0) from the Widar 3.0 dataset was randomly selected as the training set, and the activities of five participants (p1, p2, p3, p4, p5) were tested in the classroom and hall environments.

**Figure 8:**
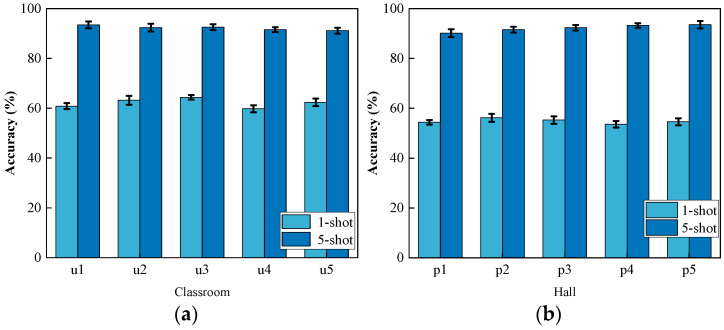
Activities performed by new users. (**a**) Our data; (**b**) Widar 3.0.

Wi-CHAR achieved the highest accuracy of 93% in the five-shot condition. The average accuracy in the "one sample per category" condition was approximately 55%. The performance difference between testing on our data and Widar 3.0 can be attributed to the number of users and types of actions. Widar 3.0 had sixteen users for testing, whereas this experiment only included six users, and there were differences in the types of actions included in the two datasets. The experimental results are depicted in Figure 8.

#### 4.2.4. Cross-User and Cross-Scene Recognition Effect

**Figure 9:**
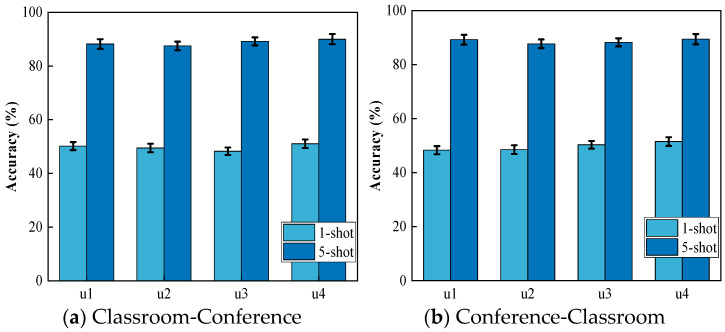
Activities performed by a new user in a new scenario.

In this set of experiments, the training and testing categories remain consistent, but both users and scenarios are altered. These experiments aim to identify the activity of a new user in a new scenario. The results of these experiments are illustrated in Figure 9. “Classroom-Conference” denotes the utilization of activity samples collected in the classroom scenario to train the Wi-CHAR system, while samples obtained from the conference room scenario are used to assess the system’s performance. For instance, “u2” represents the second user.

### 4.3. Discussion and Analysis

As observed in Section 4.2 above, the system implemented in this study demonstrates satisfactory performance under varied conditions. The recognition accuracy on our datasets is marginally higher than that of the Widar 3.0 and WiAR datasets. This discrepancy may stem from the fact that the samples in this paper are derived from data post-multi-WiFi device selection, resulting in improved data quality compared to the public datasets. Additionally, the action types examined in this paper primarily comprise common daily activities, which are coarse-grained and relatively less susceptible to environmental influence.

#### 4.3.1. Effect of the Number of Rx and Dynamic Selection

**Figure 10:**
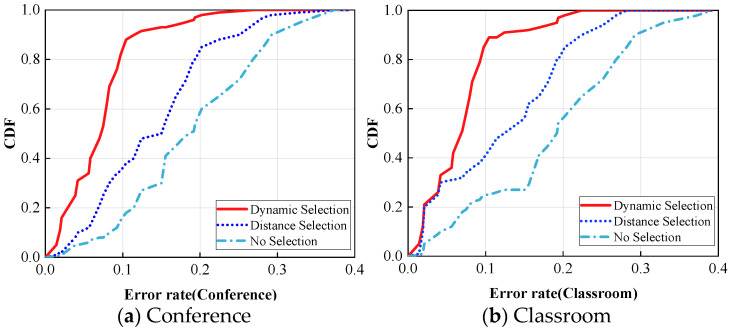
Comparison of device selection accuracy across domain conditions.

To elucidate the impact of the number of WiFi devices, the experiments in this section vary the number of Rx from two to seven (five-shot) in both the conference room and the classroom environments. Increasing the number of Rx devices leads to higher accuracy and less variation, as dynamic device selection mitigates the performance degradation caused by improper device placement. It can be observed that the improvement diminishes when the number of receiving devices exceeds five. Therefore, it can be inferred that having more WiFi devices in a typical home environment is beneficial, as long as there is sufficient space. However, when there are more than five devices, the enhancement in perceptual accuracy is not as pronounced. Each group of experiments comprises three cases of dynamic device selection (Dynamic Selection), selection by distance (Distance Selection), and no selection (No Selection), as depicted in Figure 10. Even in cross-domain scenarios, the recognition error rate of dynamic device selection remains predominantly below 0.1, which is significantly superior to non-dynamic selection.

#### 4.3.2. Effect of Different Sample Sizes

**Figure 11:**
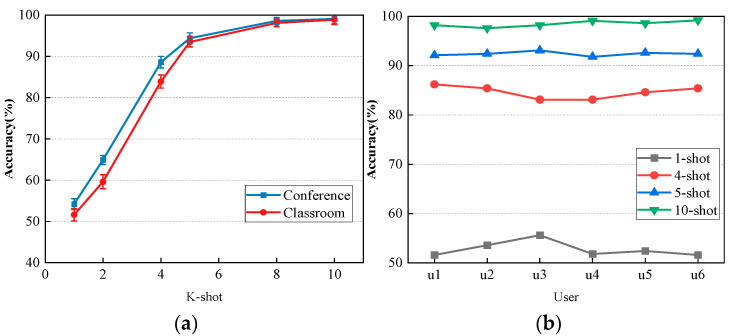
(**a**) Different sample sizes–different environments; (**b**) Different sample sizes–different user.

The experiments in this section examined the impact of different sample values on the accuracy of the Wi-CHAR platform by adjusting various K values (sample values in each category) of the training prototype network, as shown in Figure 11a. Additionally, the effect of different subjects on various sample sizes was verified, as depicted in Figure 11b.

From the aforementioned experimental results, it can be deduced that our network demonstrates minimal influence between different environments and subjects. The average accuracy exceeds 93% in the five-shot condition, while in the ten-shot condition, the average recognition rate surpasses 97%. In other words, recognition accuracy increases gradually as the number of samples increases.

#### 4.3.3. Effect of MF-DBSCAN Algorithm

**Figure 12:**
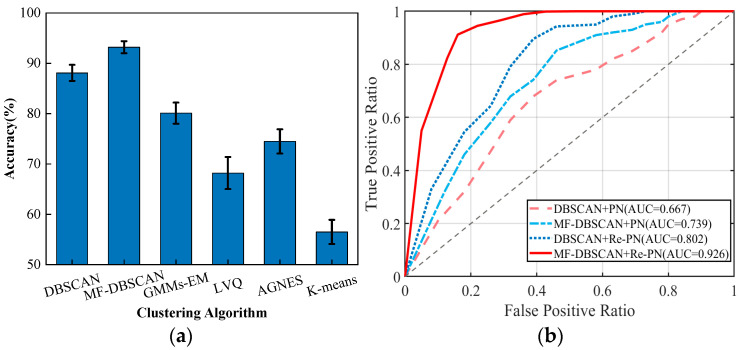
(**a**) Effect of base classifier type; (**b**) Effect of MF-DBSCAN method on classification network.

To validate the degree of impact of improved clustering-based data processing algorithms on the system, this section compares density-based clustering (DBSCAN), improved density-based clustering (MF-DBSCAN), Gaussian mixture models (GMMs-EM), K-mean clustering algorithms (K-means), learning vector quantization algorithms (LVQ), and hierarchical clustering methods (AGNES). The comparison results are shown in Figure 12a. From the comparison results, we can see that the accuracy of the traditional DBSCAN algorithm is above 85%, and the improved MF-DBSCAN algorithm can reach more than 92%, which is higher than other clustering algorithms. Therefore, the improved DBSCAN algorithm is selected for the data clustering process in this paper.

Next, we analyzed the effect of the MF-DBSCAN algorithm on the classification network used in this paper, and the experimental results in Figure 12b show that the classification model (DBSCAN + PN) with only traditional DBSCAN and traditional prototype network processing is relatively poor (the AUC is only 0.667), while the classification model using the improved DBSCAN method under the traditional prototype network condition has an AUC of 0.739 and 0.802 under the DBSCAN+Re-PN condition. The AUC of the classification model using the improved DBSCAN method under the traditional prototype network condition is 0.739, and the AUC under the DBSCAN + Re-PN condition is 0.802. It can be concluded that the improved prototype network is obvious for the classification effect of this paper, and the model advantage is significantly improved. Furthermore, the AUC under the MF-DBSCAN + Re-PN condition can reach 0.926, showing that the impact of MF-DBSCAN on the classification model is also larger. Our improvement of the two traditional methods has had a significant performance improvement.

#### 4.3.4. Comparison of Different Metrics Models

**Figure 13:**
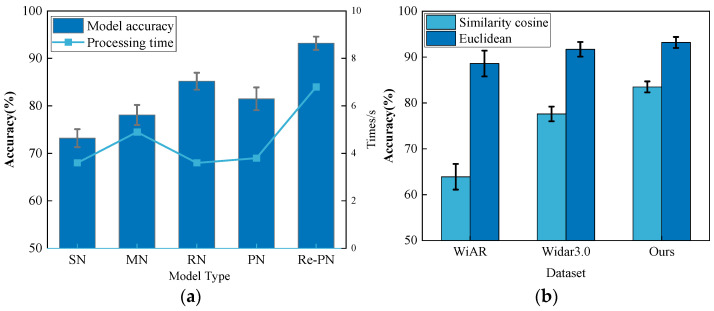
(**a**) Comparison of different similarity computational network models; (**b**) Comparison of different similarity measures.

The cornerstone of the HAR system Wi-CHAR proposed in this paper is the reassignment of a prototype network (Re-PN), an improvement upon the original PN. To assess the effectiveness of this enhancement, the experiments in this section compare the performance of the conventional PN, the Re-PN within the current system configuration, and other similar computing network structures (Siamese Network (SN), Matching Network (MN), and Relation Network (RN)). Additionally, as depicted in Figure 13a, the average accuracy of Re-PN is 12% higher than that of the traditional PN.

The choice of similarity metric is another crucial factor. This experiment compares the effects of two metrics, namely Euclidean distance and cosine similarity. The experiments in this section were conducted multiple times within the domain for three datasets, with the data input type being DFS. As illustrated in Figure 13b, the average accuracy of Wi-CHAR based on cosine similarity is lower than that of Wi-CHAR based on Euclidean distance. Therefore, it is more appropriate to employ Euclidean distance rather than cosine similarity in the Re-PN model.

#### 4.3.5. Algorithm Complexity Analysis

For Algorithm 1 and Algorithm 2, the time complexity of Algorithm 1 is $O(n^{2})$, where n denotes the number of candidate Rx’s, of which there are only a small number. For MF-DBSCAN, the basic time complexity is related to the amount of clustered data, deriving the points whose densities are connected according to eps_list, Minpts_list, and then iterating until all core sample points have a corresponding class, related to the time required to find the points, but this is of a smaller order of magnitude. The worst case is $O(m^{2})$, where m is the number of points, and its space complexity is O(m). Our feature extraction uses Resnet18 [33] and then operates by Euclidean distance metric, softmax, etc. The time complexity mainly comes from convolutional operations; the time complexity of this framework is 1.8 × 10^9^. This shows that our framework is significantly better than methods such as CNN + LSTM in terms of time overhead.

#### 4.3.6. Comparison with Existing Methods

We have compared Wi-CHAR with several other recent cross-domain recognition methods in various ways to demonstrate the performance of our approach. These include transfer learning frameworks (Sheng et al. [20]), traditional CNN/RNN frameworks (CLAR [36], CDAR [37]), adversarial learning architectures (CrossGR [38]), and meta-learning frameworks (MatNet-eCSI [28], ML-WiGR [39]). We focused on the core metrics common to the above methods: accuracy, recognition target, main algorithm, and input features, using them as benchmarks for comparison while avoiding the introduction of other presentations and parameters. Although each method achieves some degree of cross-domain effect, the Wi-CHAR method can handle multiple domain factors, such as users and environments. Despite using DFS features, the MF-DBSCAN method does not consume more time. In terms of algorithms, for the basic feature extraction model, we only used CNN, which saves more training time compared to frameworks that commonly utilize CNN + LSTM methods. Additionally, the few-shot learning method can adapt to new domains with fewer samples, while transfer learning and adversarial learning methods require additional data samples.

Wi-CHAR achieves high recognition accuracy, demonstrating that our model is robust and can achieve acceptable generalization with a small number of training samples. Further details are provided in Table 4.

## 5. Conclusions

This paper proposes the Wi-CHAR system, a WiFi-based cross-domain HAR system focusing on scenes and restricted data. It achieves high accuracy and generality in HAR over large areas with fewer samples. Wi-CHAR demonstrates robustness and versatility, delivering effective results across various scenes. It overcomes the challenge of significant degradation in model accuracy in cross-domain scenarios and eliminates the need for retraining when data acquisition in real environments is limited.

The system’s performance is evaluated in various real-life scenarios in this research. Even with a limited amount of training marker data, Wi-CHAR achieves a cross-domain average accuracy of 93.2% in recognizing human activity in a multi-WiFi link setting. This development represents progress in applying HAR technologies to smart homes, smart healthcare, and smart senior care, holding practical significance. However, further validation of our system’s performance on a broader scale is needed due to the limitations of experimental settings and experimenters. How to achieve more lightweight cross-domain activity awareness that is closer to real-life scenarios is a topic for future research. The technology also requires further enhancement and optimization for use in more realistic settings.

## Figures and Tables

**Table 1:** Types of human activity.

No.	Details
Categories	Sit, Stand, Push, Fall, Walk, Wave
Scenarios	Conference (6 m × 10 m), Classroom (10 m × 12 m)
Users	Six adults (three males, three females, height: 1.55–1.90 m, weight: 42–110 kg)

**Table 2:** Accuracy of HAR in different scenes.

Train Set	Test Set	Action Recognition Rate (%)	
		1-Shot	5-Shot
M1	M2	60.2 ± 1.2	92.3 ± 1.5
	M3	63.4 ± 0.9	93.5 ± 1.3
M2	M1	62.1 ± 0.8	92.5 ± 1.1
	M3	64.3 ± 1.3	94.1 ± 0.9
M3	M1	59.6 ± 1.8	92.8 ± 1.5
	M2	60.6 ± 1.4	92.1 ± 1.6

**Table 3:** Accuracy of HAR in different scenes (Widar 3.0).

Train Set	Test Set	Action Recognition Rate (%)	
		1-Shot	5-Shot
W1	W2	53.2 ± 1.3	89.1 ± 1.2
	W3	51.1 ± 0.8	92.1 ± 1.3
W2	W1	56.4 ± 0.9	91.2 ± 1.1
	W3	58.6 ± 0.7	92.6 ± 1.8
W3	W1	57.4 ± 0.8	90.8 ± 1.1
	W2	55.2 ± 0.7	91.5 ± 1.3

**Table 4:** Comparison of Wi-CHAR with other cross-domain systems.

Methods	Target	Features	Algorithms	Accuracy (%)
Sheng et al. [20]	4 Actions; Environment	CSI Amplitude and phase	CNN + multilayer Bi-LSTM	>90
MatNet-eCSI [28]	6 Actions; Users	Enhanced CSI	CNN + LSTM, One-Shot Learning	93.4
CLAR [36]	Actions; Locations	CSI Amplitude	Singular Spectrum Analysis, BLSTM	>86
CrossGR [38]	15 Gestures; User, Environment	CSI Amplitude	Data Augment, GAN	>82.6
CDAR [37]	6 Actions; User, Position, Direction, Environment	CSI Amplitude	CNN + LSTM, DTW, MMD	>80
ML-WiGR [39]	5 Actions; Location, Environment, Orientation, Person	DFS, BVP	CNN + LSTM, Meta-learning	>87
Wi-CHAR (Proposed)	6 Actions; User, Environment, User + Environment	DFS	CNN, Few-Shot Learning	>93

## Data Availability

The data presented in this study are available on request from the corresponding author. The data are not publicly available due to the fact that the team has not fully completed the project; therefore, the data are not disclosed for the time being.
